# A Review of Targeted Therapies for Monogenic Epilepsy Syndromes

**DOI:** 10.3389/fneur.2022.829116

**Published:** 2022-02-17

**Authors:** Vincent Zimmern, Berge Minassian, Christian Korff

**Affiliations:** ^1^Division of Child Neurology, University of Texas Southwestern, Dallas, TX, United States; ^2^Pediatric Neurology Unit, University Hospitals, Geneva, Switzerland

**Keywords:** genetic epilepsy, gene therapy, anti-sense oligonucleotide, ketogenic diet, channelopathy, molecular chaperone

## Abstract

Genetic sequencing technologies have led to an increase in the identification and characterization of monogenic epilepsy syndromes. This increase has, in turn, generated strong interest in developing “precision therapies” based on the unique molecular genetics of a given monogenic epilepsy syndrome. These therapies include diets, vitamins, cell-signaling regulators, ion channel modulators, repurposed medications, molecular chaperones, and gene therapies. In this review, we evaluate these therapies from the perspective of their clinical validity and discuss the future of these therapies for individual syndromes.

## Introduction

As of October 2021, the Online Mendelian Inheritance in Man database lists 1,285 genes or loci involved in epilepsy. Several of these discoveries have led to the development of gene-specific therapies—sometimes called “precision medicine”—ushering in a new era of gene-based therapies for epilepsy. By precision therapy or precision medicine, we mean any therapy that is either designed on the basis of the patient's underlying genetic diagnosis or which has been found through clinical trials to have a significant effect in a particular genetic epilepsy (even if the mechanism of the therapy is unknown). This broad definition of precision therapy is in contrast with an *ad hoc* treatment of seizures without any attention being paid to the genetic diagnosis.

In this review, we summarize the current state of precision therapies for monogenic epilepsy syndromes. These therapies are quite diverse, and include molecular chaperones, use or avoidance of ion channel blockade for channelopathies, repurposing of medications, diets, gene therapies including anti-sense oligonucleotides (ASO), RNA interference (RNAi), and inhibitors of over-active cellular signaling (1). While a genetic understanding of how individual patients metabolize and respond to anti-seizure medications are fast advancing (i.e., pharmacogenomics) and could be viewed as a personalized or precision therapy, these advances are outside the scope of this review (2). Throughout this review, we discuss monogenic epilepsy syndromes that are characterized by developmental and epileptic encephalopathies (DEE), according to the definition proposed by the ILAE (3).

We characterize the precision therapies by their documented efficacy as follows:

- *established*, based on randomized clinical trials having demonstrated their usefulness,
- *potential*, based on small or non-randomized clinical trials or case reports
- *hypothetical*, based on animal studies or *in vitro* data, but little-to-no clinical evidence available yet.

This review will summarize these approaches based on type (e.g., molecular chaperone, diet, gene therapy) rather than on documented success (i.e., established, potential, hypothetical), as this creates a more logical scheme for categorization. Therapies were identified through recent reviews and additional literature review for monogenic epilepsy syndromes (1, 4–6). We conclude with a discussion on the future of precision medicine in genetic epilepsies.

## Therapies for Genetic Epilepsies

### Diet and Vitamins

Specialized diets like the ketogenic diet and the modified Atkins diet have become a well-established therapeutic modality for severe seizure syndromes. Thanks to advances in our understanding of certain genetic epilepsies with underlying enzymatic or metabolic disturbances, specific diets and vitamins are increasingly becoming part of the precision medicine armamentarium (see Table 1).

**Table 1:** Diet and vitamins for monogenic epilepsy.

**Gene**	**Epilepsy syndrome**	**Suggested precision medicine**	**Therapeutic rationale**	**Status as precision medicine**
*ALDH7A1*	Vitamin B6-deficient epilepsy	Pyridoxine, lysine-restricted diet	Impairment of lysine breakdown	Established (11)
*CAD*	DEE	Uridine	Disruption of pyrimidine metabolism	Established (15)
*Folate cycle genes: FOLR-1, MTHFR, DHFR, PCFT*	Cerebral folate transporter deficiency (ataxia and refractory myoclonic epilepsy)	Folinic acid, 5-methyltetrahydrofolate	Supplementation of active metabolite missing in folate cycle	Established (13, 14)
*PIGA* ^*^	X-linked recessive multiple congenital anomalies – hypotonia – seizures syndrome (MCAHS2), epileptic encephalopathy	Ketogenic diet	Unclear	Potential (10)
*PNPO*	Vitamin B6 – deficient epilepsy	Pyridoxal-5-phosphate	Supplementation of deficiency	Established (12)
*PLPBP*	Vitamin B6 – deficient epilepsy	Pyridoxine, pyridoxal-5-phosphate	Supplementation of deficiency	Established (92)
*SLC2A1 (GLUT1)*	*GLUT1* deficiency syndrome	Ketogenic diet	Alternate energy source	Established (8, 9)

* *Indicates the current absence of a molecular or genetic rationale for this particular therapy*.

One of the best-known examples of genetic disease that responds to a specialized diet is *GLUT1* deficiency syndrome (*GLUT1-DS*), a syndrome that often provokes global developmental delay, movement disorders, and epilepsy, associated with low glucose in the cerebrospinal fluid (CSF) or hypoglycorrhachia. The hypoglycorrhachia and associated symptoms are caused by variants in *SLC2A1* which encodes the type 1 glucose transporter located in the blood-brain barrier (BBB) (7). Because the lack of CSF glucose explains the symptomatology, providing ketones to the CNS *via* the ketogenic diet provides an alternate energy source for neurons. The ketogenic diet leads to improvements not only in the seizure burden but also in the motor and cognitive symptoms in *GLUT1-DS* (8, 9).

The use of ketogenic diet for other genetic epilepsies has expanded beyond the well-known application for GLUT1-DS. Two brothers with newly-identified disease-causing variants in phosphatidyl inositol glycan A (*PIGA*) resulting in X-linked recessive multiple congenital anomalies—hypotonia—seizures syndrome (MCAHS2) who had poorly controlled seizures on multiple anti-seizure medications (ASMs) were able to achieve seizure freedom with the ketogenic diet (10). Discontinuation of the diet led to seizure recurrence. This case report was the first to note a prompt seizure response with initiation of ketogenic diet in *PIGA*-associated early-onset epileptic encephalopathy (10).

Supplementation of pyridoxine (vitamin B6) and pyridoxine derivatives is another very impactful dietary measure for specific genetic epilepsies. Pyridoxine-dependent epilepsy (PDE) is a neurometabolic disorder that presents in the neonatal period with intractable seizures that respond sometimes in dramatic fashion to pyridoxine but not to typical ASMs (7, 11). While this form of epilepsy was known as early as the 1950's, its genetic origins were only identified in 2006 when biallelic variants in *ALDH7A1*, the gene encoding *antiquitin*, were found to be responsible for this neonatal epilepsy syndrome (7). Interestingly, *antiquitin* is a dehydrogenase involved in lysine catabolism that is primarily unrelated to B6 metabolism, but its deficiency results in the accumulation of metabolites inactivate pyridoxal 5'-phosphate (PLP), which is the active form of pyridoxine that is necessary for healthy neurotransmitter function (7). Pyridoxine administration overcomes the inactivation of PLP and then normalizes the metabolic abnormality that causes neonatal seizures.

In 2005, pyridoxal phosphate-dependent epilepsy was found to be caused by bi-allelic variants in the *PNPO* gene encoding the PLP oxidase (7). *PNPO* is involved in the last step of PLP synthesis (the active form of vitamin B6). Phenotypically, patients with *PNPO* deficiency present with epileptic encephalopathy, microcephaly, and developmental delay and respond well to PLP supplementation (7). Classically, patients with *PNPO* deficiency present in infancy and their epilepsy does not respond to pyridoxine, but the phenotypic spectrum of *PNPO* deficiency is expanding as there are documented cases of *PNPO* deficiency with delayed onset or response to pyridoxine (rather than PLP), suggesting that clinicians need to keep an eye open for atypical presentations of this epilepsy (12).

Several patients with vitamin-B6 responsive epilepsy but with genetic testing negative for disease-causing variants in *ALDH7A1* and *PNPO* were found to have bi-allelic variants in the *PLPBP* gene that encodes the proline synthetase co-transcribed homolog (PROSC) which was later renamed as PLP homeostasis protein (PLPHP) (7). This protein binds to PLP and is thought to be important for mitochondrial metabolism. As genetic sequencing technologies become more common in clinical practice, the list of genetic causes of vitamin B6-responsive epilepsy is likely to continue to increase.

Cerebral folate deficiency, caused by mutations in several genes implicated in the folate cycle (notably *FOLR-1* which encodes the cerebral folate receptor), manifests as developmental regression with ataxia, choreoathetoid movements, and myoclonic epilepsy starting around age 3 (13). CSF analysis shows low levels of 5-methyltetrahydrofolate (5-MTHF, <5 nmol/L) but normal serum levels of that metabolite (13). The condition responds very well to high-dose folinic acid, making it important to make the correct diagnosis. Other folate derivatives, like 5-methyltetrahydrofolate, have also been used with some success (14).

Lastly, a relatively new genetic epilepsy syndrome caused by an inborn error of metabolism for which dietary supplementation acts as a precision medicine has been reported recently. Researchers were able to identify four children across three families who presented to medical care for epileptic encephalopathy, global developmental delay, and anemia with anisopoikilocytosis (15). They were able to identify biallelic variants in *CAD*, a gene encoding a multifunctional enzyme involved in *de novo* pyrimidine biosynthesis, as the likely cause of this neurometabolic disorder (15). Two of the children had a worsening neurodegenerative course resulting in death at ages 4 and 5 respectively, but the remaining two children were treated with oral uridine supplementation (which allows recycling of pyrimidines) and showed significant developmental progress (15). The authors suggest that there may be an indication for adding *CAD* to the list of genetic conditions on the newborn screening as early detection of the disease could significantly impact the course of the illness.

### Inhibiting Overactive Cellular Signaling

Several genetic epilepsy syndromes are the result of mutations in genes that regulate cellular proliferation. Medications that inhibit cellular overgrowth are rational drug candidates for these conditions and were found to be quite effective as adjunctive treatments (see Table 2). *TSC1/2* mutations, result in hyperactivity of mTOR complex 1 (mTORc1) with mTOR being a key regulator of cell growth and survival. The hyperactivity produces abnormal neuronal differentiation and migration. Up to 90% of patients with tuberous sclerosis from TSC1/2 mutations will develop epilepsy, with two-thirds of cases being medication-resistant (16). Everolimus, an mTOR inhibitor originally approved for the treatment of renal angiomyolipomas and TSC-associated subependymal giant cell astrocytomas, was subsequently approved as adjunctive therapy for TSC-associated focal seizures in children greater than age 2 (16). The randomized, double-blind, placebo-controlled phase 3 EXIST-3 study supported the use of everolimus as an adjunct if seizures could not be controlled with two ASMs (17). There is ongoing work to determine whether the indication for this drug should be extended to children younger than age 2, as some studies suggest a benefit for the infant age group (18).

**Table 2:** Inhibitors of cellular signaling.

**Gene**	**Epilepsy syndrome**	**Suggested precision medicine**	**Therapeutic rationale**	**Status as precision medicine**
*GATOR1 complex (DEPDC5, NPRL2, NPRL3)*	Familial focal epilepsy with variable foci	mTOR inhibitors (everolimus)	Inactivation of mTOR pathway	Potential (1, 93)
*GNAQ*	Sturge-Weber-related epilepsy	mTOR inhibitors (sirolimus)	Inactivation of mTOR pathway	Potential (27)
*PIK3CA*	Intractable epilepsy	PI3K inhibitors	Suppression of PI3K signaling	Potential (29)
*TSC1, TSC2*	Tuberous sclerosis, focal cortical dysplasia	mTOR inhibitors (sirolimus, everolimus, 1,3,5-triazine derivatives)	Inactivation of mTOR pathway	Established (19)

Much of the research effort for mTORopathies has focused on everolimus but other mTOR inhibitors, specifically rapamycin or sirolimus, have also been studied (19). A mouse model of TSC with *Tsc1* conditional inactivation primarily in glia exhibits progressive epilepsy and premature death. Early administration of rapamycin in *Tsc1*-inactivated mice prevented the development of epilepsy and premature death compared to the untreated mice, while late administration suppressed seizures in mice that had already started having seizures and also prolonged survival (20). The efficacy noted in this animal model has also been documented in two pediatric case reports. The first reported on a 10 year-old girl who experienced a dramatic decrease in seizure frequency after 10 months of rapamycin therapy (21). The second case report documented significant improvement in seizure frequency in eight children with TSC during the 1st year but with worsening of seizure frequency in three of those patients (22). After discontinuation of rapamycin, three of five children experienced recurrence of seizures, suggesting an overall benefit at least for the 1st year (22).

In addition to case reports, two studies thus far have evaluated the role of sirolimus in TSC-related epilepsy. An open-label study of sirolimus in children with TSC-related epilepsy (23) found that sirolimus treatment led to a statistically insignificant 41% decrease in seizure frequency compared to the standard-of-care. This result is in contrast with a larger pediatric study of sirolimus in TSC-related epilepsy that found a statistically-significant 78% decrease in seizure frequency, 47% of whom went on to be seizure-free (24). Subgroup analysis of patients with drug-resistant seizures also confirmed a statistically significant improvement in seizure control (24). Sirolimus may therefore become an important alternate for patients unable to tolerate everolimus. There are no randomized trials comparing sirolimus to everolimus but a retrospective multicenter study of patients with TSC-related epilepsy suggests that there were more adverse events and dosing issues with sirolimus use compared to everolimus (25). There are also newer 1,3,5-triazine derivatives under development that have shown promise as mTORc1/2 inhibitors as they have excellent tolerability profiles and demonstrate marked suppression of spontaneous recurrent seizures in two mouse models of epilepsy, including a mouse model of TSC-related epilepsy (26).

Other disorders of cellular proliferation—including Sturge-Weber syndrome caused by mutations in *GNAQ, NPRL3*-related cortical malformations, and *PIK3CA*-related overgrowth syndromes—also present with epilepsy that seems responsive to “rapalogs” like sirolimus and everolimus. A retrospective observation study of six patients with refractory epilepsy from Sturge-Weber syndrome reported complete seizure control in all patients with minimal side-effects (27). A case report of a neonate with NPRL3-related epilepsy reported 3.5 months of seizure control that allowed the patient to grow seizure-free until epilepsy surgery (28). Mutations in the catalytic subunit of phosphoinositide 3-kinase (*PIK3CA*) are associated with a phenotypic spectrum of bilateral dysplastic megalencephaly and focal cortical dysplasia causing pediatric epilepsy. In a mouse model of *PIK3CA*-related epilepsy expressing the most common human mutations and which recapitulates the human phenotype, a new inhibitor of PI3K signaling that is being trialed for solid tumors (BKM120, a 2,6-dimorpholino pyrimidine derivative) significantly increased the seizure threshold (29). While these studies are promising, there is an ongoing need for studies of mTOR inhibitors for other mTOR-opathies involving mutations in related genes such as the GATOR complex (*DEPDC5, NPRL2*).

### Ion Channel Modulators

Many genetic epilepsies stem from mutations in genes encoding voltage-gated ion channels and are generally referred to as “channelopathies.” This section will discuss the use of ion-channel modulators for these genetic epilepsies, specifically sodium and potassium channelopathies (see Table 3). It should be noted that precision therapies other than ion-channel modulation have also been developed for these channelopathies and these are covered in their respective sections.

**Table 3:** Precision therapies for channelopathies.

**Gene**	**Epilepsy syndrome**	**Suggested precision medicine**	**Therapeutic rationale**	**Status as precision medicine**
*KCNA2*	DEE	4-aminopyridine	Reducing current amplitudes	Potential (43)
*KCNQ2*	DEE	Sodium channel blockers, retigabine, gabapentin	Selective potassium channel Kv7 opener (retigabine), potassium channel Kv7 activator (gabapentin)	- Sodium channel blockers – established (44) - Retigabine – potential (47) - Gabapentin – potential (48)
*KCNT1*	Epilepsy of infancy with migrating focal seizures, nocturnal frontal lobe epilepsy	- Quinidine - ASO	- Potassium channel blockade in GOF variants - Gene silencing	- Quinidine – potential (7, 49) - ASO – potential (86)
*PRRT2*	Benign familial infantile epilepsy, paroxysmal kinesigenic dyskinesia	Sodium channel blocker	Failure of neurotransmission	Potential (64, 65)
*SCN1A*	Dravet syndrome	- Avoid sodium channel blockers - Stiripentol - Fenfluramine - Cannabidiol - ASO	Loss of function of NaV1.1 sodium channels	- Avoidance of sodium channel blockers – established (1, 6) - Stiripentol – established (35) - Fenfluramine – potential (36) - Cannabidiol – established (16, 37) - ASO – hypothetical (84)
*SCN2A*	Ohtahara syndrome, early encephalopathy	Sodium channel blockers	Gain of function of NaV1.2 channel	Potential (31)
*SCN8A*	DEE	- Sodium channel blockers - ASO		- Sodium channel blockers – potential (42) - ASO – hypothetical (85)

#### Sodium Channelopathies

In order of population prevalence, *SCN1A, SCN2A*, and *SCN8A* are the four voltage-gated sodium channel genes that are most commonly associated with epilepsy (30–32). We will first consider therapies for *SCN1A* before addressing precision therapies for *SCN2A/8A*-related epilepsy because similar considerations regarding sodium channel blockade apply to these three conditions (7).

*SCN1A* mutations are associated with various forms of seizures and epilepsies on a wide spectrum of severity, including “isolated” febrile seizures as well as Dravet syndrome (DS), a developmental and epileptic encephalopathy of poor prognosis. Eighty percent of DS cases are associated with loss-of-function (LOF) mutations in *SCN1A* that encodes the voltage-gated sodium channel Nav1.1, particularly in inhibitory interneurons (33). Precision therapy for DS involves avoidance of sodium channel blockers (e.g., carbamazepine, lamotrigine), as they can worsen symptoms in some patients, as well as the use of agents that enhance GABAergic neurotransmission (e.g., clobazam) (33, 34). Typically, seizures remain refractory to multiple drug combinations, and no single therapeutic approach has shown long-term efficacy in these patients, up to now. Though not yet developed for clinical use, a peptide derived from spider venom (Hm1a) seems promising as it selectively activates the Nav1.1 channel with consequent improvement in seizure and mortality outcomes in a mouse model of DS (33). Other precision therapies for DS [i.e., stiripentol, fenfluramine, and cannabidiol (35–37)] will be discussed in the next section on repurposed medications.

Disease-causing variants in *SCN2/8A* present in a very heterogenous manner that ranges broadly from developmental delay without epilepsy to severe early-onset DEE. Adding to this complexity, individuals with identical variants can present very differently (31, 32, 38). Despite this diversity of clinical presentations, a pattern seems to be emerging which suggests that patients with gain of function (GOF) variants exhibit the more severe early-onset epileptic encephalopathies (EE) while patients with LOF variants present later in life with autism or developmental delay (7, 39–41). This general rule about GOF vs. LOF variants will certainly need further studies to establish its validity, but for the time being it provides clinicians with a rationale for the current management of these sodium channelopathies [e.g., the use of sodium channel blockers like high-dose phenytoin for patients with early-onset *SCN8A* epilepsy (42)]. When it comes to the sodium channelopathies as a whole, their clinical response to sodium channel blockers remains an important area of research.

#### Potassium Channelopathies

4-aminopyridine is a potassium channel blocker that can antagonize GOF defects in the *KCNA2* gene that cause a DEE. In a study of n-of-1 trials in nine different centers, nine of 11 patients showed improvement in seizure burden, gait, ataxia, alertness, and cognition after starting 4-aminopyridine (43). Because of these findings, it seems a promising tailored treatment for KCNA2-encephalopathy caused by GOF variants.

Heterozygous variants (usually frameshifts or deletions) in *KCNQ2*, which encodes the alpha subunit of the potassium channel Kv7.2, underlie the majority of the autosomal dominant cases of benign familial neonatal epilepsy (BFNE). Variants in this gene associated with DEE are more commonly missense variants with a dominant negative effect (44). Most of these variants seem to induce loss of function of the voltage-gated potassium channel. Sodium channel blockers, which are not a form of precision medicine for this condition, have shown some efficacy, specifically for BFNE (45). Molecular insights into *KCNQ2* disease-causing variants are stimulating research into targeted therapies. Ezogabine (EZO), for example, increases the opening of *KCNQ2* channels, providing a rationale for its use in *KCNQ2* variants that decrease potassium channel activity (44). In 11 patients treated with EZO, seizure reduction and improvements in development were noted in three out of four patients treated before 6 months, and two out of seven patients treated after that age (44). Much like EZO, retigabine and gabapentin have been shown *in-vitro* to be selective Kv7 openers and seem poised to be used for *KCNQ2*-related epilepsy (46, 47). For example, initiation of gabapentin led to rapid and sustained improvement in seizures in a patient with *KCNQ2* DEE (48). Retigabine is an ASM that has been pulled from the market due to significant side-effects but had shown significant promise for refractory epilepsy – there is cause for optimism as new retigabine analogs with fewer side-effects are under development (47).

Pathogenic variants in *KCNT1*, also encoding a potassium channel, are responsible for a broad phenotypic spectrum that include autosomal dominant nocturnal frontal lobe epilepsy (ADNFLE), early-onset epileptic encephalopathy (EOEE), and epilepsy of infancy with migrating focal seizures (EIMFS) in neonates and infants, which are often refractory to conventional ASMs (49). Initial studies suggested that *KCNT1*-related epilepsy caused by a GOF variant responds to quinidine, a sodium and potassium channel blocker mostly used as an anti-arrhythmic (50). However, subsequent clinical experience found that it was mostly ineffective in early-onset EE (51). A subsequent study of patients with *KCNT1*-related epilepsy noted a > 50% seizure reduction in 20% of patients, and with only a few achieving transient seizure freedom (49). While quinidine has had mixed results for this monogenic epilepsy syndrome, its robust efficacy in patients with specific variants in *KCNT1* does give hope that it may become standard therapy for those specific variant (49). Till then, *KCNT1* serves as an important reminder that, despite strong biomolecular evidence suggesting a certain therapy (e.g., ion channel modulation), the road to a successful therapy can sometimes be more complex than initially expected.

### Repurposing Established Medications

As mentioned in the previous section, quinidine is an anti-arrhythmic agent that is being put to novel therapeutic use in epilepsy, an example of a repurposed drug. In this section, we discuss additional repurposed medications as examples of targeted therapies for monogenic epilepsy (see Table 4).

**Table 4:** Repurposed medications for monogenic epilepsies.

**Gene**	**Epilepsy syndrome**	**Suggested precision medicine**	**Therapeutic rationale**	**Status as precision medicine**
*ARX* ^*^	Epileptic-dyskinetic encephalopathy	Valproic acid, estradiol	Unclear	Hypothetical (94, 95)
*CACNA1A*	Absence epilepsy with ataxia, DEE	- Aminopyridine (LOF) - Flunarizine (GOF)	Compensation in synaptic transmission (aminopyridine) calcium channel blockade (flunarizine)	Aminopyridine, flunarizine – potential (96)
*CHRNA4/B2/A2*	Sleep-related hypermotor epilepsy	Nicotine	Desensitization of nicotinic acetylcholine receptors	Established (66)
*FRRS1L* ^*^	DEE	Sulthiame	Unclear	Potential (67)
*GABRB3*	Lennox-Gastaut syndrome	Vinpocetine	Sodium channel modulation	Potential (68)
*GABRG2*	EE	Stiripentol	Increase GABA-A receptor activity	Hypothetical (97)
*GRIN1/2A/2B/2D*	Epilepsy with centrotemporal spikes, Landau-Kleffner syndrome, DEE	- NMDA receptor antagonists (memantine, dextromethorphan) - Ketamine (GOF) - Serine (LOF)	Modulation at the NMDA receptor	Potential (62, 63, 98)
*PCDH19* ^*^	DEE	- Ganaxolone - Stiripentol	- Compensation for altered steroidogenesis - Unclear	- Ganaxolone – potential (57, 99) - Stiripentol – potential (61)
*SLC13A5* ^*^	DEE	Stiripentol	- Unclear	Hypothetical (100)

* *Indicates the current absence of a molecular or genetic rationale for this particular therapy*.

#### Dravet Syndrome

We have previously discussed the avoidance of sodium channel blockade in DS. Fenfluramine (36), cannabidiol (37), and stiripentol (35) have all been found to provide clinical benefit for this severe epilepsy syndrome in randomized clinical trials (52). These clinical findings are buttressed by animal studies, including a study of fenfluramine and norfenfluramine in a zebrafish model of DS (53) and a study of cannabidiol and stiripentol in hyperthermia-induced seizures in a mouse model of DS (54). We include these medications as “precision therapies” given their efficacy, even though the precise mechanism of action in DS patients is not fully understood. Fenfluramine was initially marketed as an appetite suppressant, typically in combination with the monoamine oxidase inhibitor phentermine. It was pulled from the market in the 1980's due to concerns of valvular heart disease and pulmonary hypertension (16). However, subsequent studies have demonstrated its safety in DS patients (36, 55, 56). Regarding cannabidiol, there is a need for independent studies as the landmark study of this drug in DS was financed by GW pharmaceuticals (37).

#### Protocadherin 19 Female Epilepsy

Protocadherin 19 female epilepsy (PCDH19-FE) is an increasingly reported form of epilepsy whose pathophysiology is poorly understood. Disease-causing variants in *PCDH19* lead to early-onset DEE with clustering epilepsy (CE) as well as altered steroidogenesis and nuclear-hormone-related gene expression changes. The known involvement of hormonal pathways in the pathogenesis of PCDH19-related epilepsy has prompted researchers to look for steroid-based treatments for this disorder. Results for corticosteroid therapy are mixed (57–59). Because patients with PCDH19-FE have been found to have lower levels of allopregnanolone, it has been theorized that replacement of that hormone with a synthetic analog, ganaxolone, which acts as a human neurosteroid, could be therapeutic (60). Stiripentol has also been found to be beneficial as an adjunctive ASM in PCDH19-FE, as documented in a case report of a young girl who became seizure-free for an unprecedented 2 years and 10 months after starting stiripentol as an add-on to valproate and clobazam (61).

#### GRIN-Related Epilepsy

Mutations in *GRIN1/2A/2B/2D* are associated with childhood-onset epilepsy and developmental delay. Depending on the resulting change in the protein structure of the N-methyl-D-aspartate receptor (NMDAR), certain mutations cause increased charge transfer while other variants reduce current. Case reports have suggested a role for NMDAR blockade with dextromethorphan (typically used in cough syrup) or memantine (used for Alzheimer's dementia) for variants that cause gain-of-function (62), while a single case report suggests L-serine supplementation for loss-of-function variants (63). It should be noted that functional changes of receptor function (e.g., NMDA and GABA) or ion channel function, as determined with *in vitro* techniques or *in-silico* approaches, may end up being more relevant for the development of therapies than the current GOF/LOF dichotomy.

#### PRRT2-Related Epilepsy

*PRRT2*-related epilepsy provides a good example of repurposing drugs that had already been proved effective for a movement disorder caused by the same gene. *PRRT2* disease-causing variants are among the most common genetic causes of epilepsy. *PRRT2* encodes a pre-synaptic transmembrane protein that enables synaptic vesicle fusion (7). Mutations in *PRRT2* are associated with a broad clinical spectrum with three major phenotypes that include self-limited familial infantile seizures, paroxysmal kinesigenic dyskinesia (PKD), and infantile convulsions with choreoathetosis (64). Precision medicine therapy for *PRRT2*-related seizures with carbamazepine came from the common genetics shared between PKD and the infantile seizures. PKD had been treated effectively for years with carbamazepine and once it was understood that *PRRT2* variants explained both PKD and familial infantile seizures, carbamazepine and oxcarbazepine were used effectively for *PRRT2*-related infantile seizures (7, 65).

#### Miscellaneous Syndromes

In the last chapter of this section, we discuss a few monogenic epilepsy syndromes for which there is limited evidence (e.g., case series or reports) for certain repurposed medications. Autosomal dominant sleep-related hypermotor epilepsy caused by mutations in CHRNA4 (cholinergic receptor, nicotinic, and alpha polypeptide 4) responds strikingly well to transdermal nicotine patches in a pediatric case series (66). Hypermotor seizures disappeared entirely, but sporadic arousals persisted. Ferric chelate reductase 1 like (*FRRS1L*) encephalopathy is a rare cause of DEE with only a few cases reported worldwide and with mostly drug-refractory seizures. A case report of a Malaysian child with *FRRS1L* encephalopathy documented excellent seizure response to sulthiame, a central carbonic anhydrase inhibitor used widely in Europe and Asia but not commonly used in North America (67). A woman with Lennox-Gastaut syndrome (LGS) from a disease-causing variant in *GABRB3* experienced a sustained, dose-dependent decrease in epileptiform activity on EEG after starting vinpocetine, a synthetic derivative of vincamine which is an alkaloid derived from the periwinkle plant. Its antiepileptic properties are likely from a combination of sodium channel modulation and GABA-A activity potentiation—future studies will be needed to determine its efficacy and pharmacokinetics in epilepsy (68).

### Molecular Chaperones

Neurologists are very familiar with disorders of protein folding and aggregation, such as Huntington's disease and Alzheimer's disease, but may not be aware that certain genetic causes of epilepsy are also conformational disorders. For these genetic epilepsies, chemical chaperones—small molecules that correct these folding and aggregating abnormalities—represent an important avenue for precision medicine. These compounds work in a myriad of ways, including stabilization of misfolded proteins, reducing aggregation, preventing deleterious interactions with other proteins, and modifying the activity of endogenous chaperones to promote more efficient folding and translocation of proteins to the appropriate intracellular or extracellular destinations (69) (see Table 5). Recent examples of chemical chaperones for genetic epilepsies include studies of *Munc18-1* related epilepsy and *LGI1*-related epilepsy, which we will discuss next.

**Table 5:** Molecular chaperone treatments.

**Gene**	**Epilepsy syndrome**	**Suggested precision medicine**	**Therapeutic rationale**	**Status as precision medicine**
*LGI1*	Familial temporal lobe epilepsy	Phenylbutyrate, only for secretion-defective mutations	Molecular chaperone	Hypothetical (71)
*STXBP1*	Ohtahara syndrome, West syndrome	Phenylbutyrate in specific missense mutations and possibly LOF mutations	Molecular chaperone	Hypothetical (70)

Heterozygous *de novo* mutations in *STXBP1*, which encodes *Munc18-1*, result in a syndrome consisting of epilepsy, intellectual disability and movement disorders that carries a poor prognosis (70). A recent study in yeast, worm, and mouse neurons has shown that several disease-linked missense mutations in this gene lead to the disease phenotype through a dominant-negative effect whereby the mutant protein becomes destabilized and forms aggregates that then deplete the functional levels of wildtype *Munc18-1* protein by co-aggregation (70). Use of chemical chaperones (4-phenylbutyrate, sorbitol, and trehalose) reversed the deficits caused by the mutations, both *in-vitro* and *in-vivo* in these animal models (70). Because this approach stabilizes the remaining wild-type Munc18-1 protein, the authors of the study predict that this strategy will also work for not only missense mutations but also non-sense and truncation mutations, suggesting a possible path forward for treating the multiple genetic causes of *Munc18-1*-related epilepsy (70).

Mutations in *LGI1*, which encodes a neuronally-secreted protein, cause autosomal dominant lateral temporal lobe epilepsy (ADLTE). Yokoi et al. classified 22 reported missense mutations in *LGI1* as either leading to defects in protein secretion (secretion-defective) or allowing proper secretion (secretion-competent), and then generated two mouse models of ADLTE encoding mutant proteins that were representative of the two groups (71). The secretion-defective mouse model expressed *LGIE383A* protein, which was found to be prematurely degraded by the endoplasmic-reticulum (ER) quality-control machinery. The secretion-competent mouse model expressed *LGI1S473L* protein, which dimerized abnormally and was defective in binding to ADAM22, one of its known receptors. These two mutations resulted in loss of function (LOF) through compromised intracellular trafficking or ligand activity of LGI1 to ADAM22. Use of 4 phenylbutyrate, a chemical chaperone, restored LGI1E383A's ability to fold and bind to ADAM22 and improved the seizure susceptibility of the LGI1E383A model mice but not the LGI1S473L mice. In addition to identifying *LGI1*-related epilepsy as a conformational disease, this study suggests a bright future for chemical chaperones as a precision therapy for certain monogenic epilepsies.

### Gene Therapies

Gene therapy aims to alleviate disease by introducing genetic material into target cells to restore proper physiologic function (72). For monogenic epilepsy syndromes, gene therapies target neurons in the central nervous system (CNS), which requires either intrathecal delivery to bypass the BBB or systemic administration of the therapy that crosses the BBB to then reach the entire brain. Most gene therapies are packaged in adeno-associated viruses (AAV) that have tropism to the brain, typically AAV9 because of its BBB-permeable capsid. However, while being a prime delivery vector for gene therapies, viral capsid size leads to a limitation in terms of DNA cargo (<4.7 kB).

In addition to multiple delivery methods, there are also multiple approaches to gene therapy that we could subdivide loosely into gene editing, gene supplementation, and gene expression modification. We will discuss each of these approaches and the associated monogenic epilepsies that have been tackled using each approach (see Table 6). The details of each gene therapy are beyond the scope of this review, and the reader is directed to excellent reviews on each therapy (73).

**Table 6:** Gene therapies for monogenic epilepsies.

**Gene**	**Epilepsy syndrome**	**Suggested precision medicine**	**Therapeutic rationale**	**Status as precision medicine**
*CDKL5*	DEE	- AAV-mediated gene therapy - Fenfluramine - GSK3B-HDAC dual inhibitor - GABA receptor antagonist	- Genetic repair - Serotonin release (fenfluramine) - Histone deacetylase activity (GSK3B) - Decrease of excessive GABAergic transmission	Hypothetical (75, 101–104)
*CSTB*	Unverricht-Lundborg disease (progressive myoclonic epilepsy)	ASO	Restore normal gene splicing pattern	Hypothetical (88)
*DNM1*	DEE	AAV-mediated microRNA	RNA interference	Hypothetical (89)
*GYS1*	Lafora disease	ASO	Downregulation of glycogen synthase	Hypothetical (87)
*KCNA1*	Temporal lobe epilepsy	CRISPRa	CRISPRa-mediated upregulation of Kv1.1 channels	Hypothetical (105)

#### Gene Editing

Gene editing methodologies, the most prominent of which is the CRISPR/Cas9 gene editing system, allow for direct correction of a genetic defect. Briefly, CRISPR/Cas9 is a gene editing approach in which DNA sequences called CRISPRs (Clustered Regularly Interspaced Short Palindromic Repeats) allow the targeting and destruction of specific DNA targets by Cas (CRISPR-associated) proteins (74). CRISPR/Cas9 technology may be particularly important for genetic epilepsies since dominant heterozygous missense mutations or small insertion-deletions make up a large percentage of the known pathogenic variations. These variants make appealing targets for correction with CRISPR/Cas9 (74), but therapies have yet to emerge for clinical use.

#### Gene Supplementation

Gene supplementation usually involves the insertion of a supplemental transgene into a cell to make up for a LOF genetic defect, but does not correct the underlying defect *per se*. This approach has been trialed for cyclin-dependent kinase-like 5 (*CDKL5*) deficiency disorder, which is an X-linked dominant disorder caused by *de novo* mutations in *CDKL5* leading to a DEE. A gene supplementation study involving AAV delivery of the human *CDKL5* gene in *CDKL5* knock-out mice demonstrated improved behavior and restoration of synaptic function in neurons (75). While the study was limited (e.g., incomplete recapitulation of the human phenotype including seizures, weak effect of treatment on some parameters), it serves as a proof-of-concept for gene supplementation strategies for *CDKL5*-related epilepsy (72, 75).

Similar work has been done in animal models of tuberous sclerosis complex. The proteins hamartin and tuberin function within a complex to inhibit mammalian target of rapamycin (mTOR)-mediated cell growth and proliferation. Mutations in *TSC1* or *TSC2* (hamartin and tuberin, respectively) lead to tuberous sclerosis complex, a tumor suppressor syndrome characterized by overgrowth in multiple organs including the brain, resulting in intellectual disability, autism, and epilepsy. Several gene supplementation strategies have shown promise in mouse models of TSC. A mouse model of TSC with neuron-specific hamartin loss showed marked improvement in survival, weight, gain, and motor behavior after intracerebrovascular (ICV) injection of an AAV expressing tagged form of hamartin (76). Similar results were obtained with an IV. Follow up work by the same team showed that similar improvements in both phenotype and histology could be achieved through intravenous (IV) injection as opposed to ICV injection (77). Similar gene therapy efforts in a mouse model of TSC2, using AAV-mediated delivery of a “condensed” form of human tuberin, also showed significant increase in mean survival and reduction in histological evidence of brain pathology (78). These studies, taken as a whole, demonstrate the significant potential of AAV-mediated gene supplementation for TSC1 and TSC2.

Gene supplementation strategies have also been tested in various ion channelopathies that lead to epilepsy. As mentioned in a previous section, DS is mostly caused by LOF mutations in *SCN1A*, a gene that encodes the α subunit of the voltage-gated sodium channel Nav1.1 (72). As a result, most gene therapy efforts for DS have centered on increasing Nav1.1 expression. These efforts have been stymied by at least two factors. The first is that the *SCN1A* gene (6 kB) exceeds the packaging limit of AAV vectors. The second is that the proclivity to seizure in this syndrome seems to be caused by an inhibition-excitation imbalance from loss of *SCN1A* expression primarily in interneurons—restoration of healthy gene function would therefore have to specifically be targeted toward this specific cell population (79).

While the α subunit exceeds the packaging limit, the β1 multifunctional sodium channel auxiliary subunit does not exceed the limit. Moreover, the β1 subunit has been shown to increase ion flow through the α subunit and promotes the trafficking of α subunits from an intracellular pool to the cell surface. Overexpression of *NaV*β*1* should in theory improve the function of residual voltage-gated channels and thereby improve the DS phenotype. A murine study evaluated the effects of a bilateral ICV injection of an AAV vector coding for a truncated mouse promoter based on a GABAergic neuron expressing gene called *Gad-1* and mouse *NaV*β*1*, with appropriate control mice (80). Treatment led to a partial rescue of the disease phenotype that was sexually divergent (80). The moderate efficacy of the treatment may have been due to the moderate specificity of the chosen promoter for GABAergic neurons—however, this study did provide a proof-of-principle for future treatments (72).

#### Gene Expression Modification

Gene expression modification seeks to increase or decrease the expression of a gene product, also known as activation and inhibition, respectively. There are various techniques that fall under this category including anti-sense oligonucleotides (ASO) (81), RNA interference (RNAi) (82), and dCas9-based CRISPRa/i (83).

##### Anti-sense Oligonucleotides

ASOs are short, single-stranded oligodeoxynucleotides, usually 8–50 nucleotides in length, which bind to a target mRNA *via* complementary base pairing. The base pairing then leads to endonuclease-mediated transcript knockdown and decreased expression of the associated deleterious protein (81). First-generation ASOs were limited by rapid turnover (i.e., RNA degradation) and insufficient intracellular concentration to impact target genes. Modifications to their chemical backbones led to 2nd and 3rd generation ASOs that, in addition to targeting mRNA, could also bind non-coding RNAs and toxic RNAs, leading to a much wider range of clinical uses. Their main limitation remains the need for intrathecal administration as they do not cross the BBB (81).

ASOs are being used in animal models of seizure disorders related to ion channelopathies. DS was discussed in a previous section and is predominantly caused by mutations in *SCN1A*. Expression of *SCN1A* is mediated by an anti-sense non-coding RNA (SCN1ANAT). Upregulation of the healthy *SCN1A* allele using oligonucleotide-based compounds (AntagoNATs) to target SCN1ANAT in a knock-in mouse model of DS and in a non-human primate led to significant improvements in seizure phenotype in the animals (84). AntagoNATs may therefore become a helpful treatment paradigm for DS, though will likely limited by the weekly intra-cerebral administration that is required (72).

GOF mutations in the *SCN8A* gene encoding Nav1.6 results in DEE (85). Reduction of the levels of *Scn8a* transcript to 25–50% of typical expression levels using an ASO resulted in delayed seizure onset and increased survival in mouse models of both *SCN8A* encephalopathy and DS (85). This success in animal models will pave the way for further studies of ASOs to decrease the pathological increase in transcript expression for GOF mutations in *SCN8A* and *SCN2A*. LOF mutations in *SCN8A* and *SCN2A* resulting in pathological decrease in transcript expression would benefit from a CRISPRa strategy, to be discussed in a subsequent section (72).

A similar ASO strategy has been developed for GOF variants in *KCNT1* that lead to epilepsy of infancy with migrating focal seizures (EIMFS) (86). A single ICV injection of a *Kcnt1* gapmer (i.e., short DNA strands flanked by strands of RNA mimics) ASO in a mouse model of KCNT1-epilepsy led to significant reduction in seizure frequency and increased overall survival compared to mice treated with a control (i.e., non-hybridizing) sequence (86). These results suggest a promising road ahead for ASO-based therapies in *KCNT1*-associated epilepsy.

Lafora disease is a fatal, genetic progressive myoclonic epilepsy from mutations in *EPM2A* and *NHLRC1* resulting in abnormal branching patterns in a subgroup of glycogen molecules. These abnormal glycogen molecules then precipitate and accumulate as Lafora bodies that then generate a neuroinflammatory response and neurodegeneration. Glycogen synthase, encoded by the *GYS1* gene, is the enzyme responsible for glycogen branch elongation. While mutations in *GYS1* are not responsible for Lafora disease, down-regulation of glycogen synthase with an ASO could lead to decreased elongation of glycogen polymers, thereby decreasing the formation of Lafora bodies (87). ICV injection of an ASO targeting the mRNA of brain-expressed *GYS1* in a murine model of Lafora disease led to inhibition of further accumulation of Lafora bodies in older mice that had already formed bodies and prevented Lafora body formation in young mice that had not yet formed any (87). The inhibition of Lafora body formation correlated strongly with improvements in neuroinflammatory markers, suggesting all-in-all that this approach could prevent and stop the progression of this currently fatal form of epilepsy that strikes otherwise-healthy teenagers (87).

Mutations in the cystatin B gene (CSTB), which encodes an inhibitor of several lysosomal cathepsins, result in Unverricht-Lundborg disease (ULD), a form of progressive myoclonic epilepsy with limited pharmacological treatments (88). ASO-based therapies uniquely designed for particular CSTB mutations seem to be on the horizon. An ASO therapy for a newly-identified splicing mutation causing ULD restored the normal splicing pattern in a dose-specific manner in cultured cells from the affected patient, providing proof-of-principle for mutation-specific anti-sense therapy in ULD and similar genetic epilepsies (88). It should be noted, however, that the most common mutation causing ULD is an unstable expansion of a dodecamer repeat in the promoter region, leading to down-regulation of *CSTB* mRNA levels (88), and the remaining mutations are missense, non-sense, frameshift, and splice-site.

Before moving to a discussion of other gene therapy strategies, it should be noted that, in addition to the challenge of intrathecal administrations of the gene product mentioned previously, another limitation of ASOs is the need for multiple administrations per year—in contrast with the one-time administration of gene supplementation strategies.

##### RNA Interference

RNA interference (RNAi) has also emerged as an important player in the modification of gene expression for therapeutic purposes. In most cases, RNAi is used to silence dominant toxic genes in an allele-specific way, meaning that the technology ideally silences the toxic allele while allowing healthy expression of the normal allele (82). Briefly, RNAi refers to a process whereby double-stranded RNA (dsRNA) is processed into small-interfering RNAs (siRNAs) by the ribonuclease-III (RNase-III) enzyme called Dicer. Measuring about 21–23 nucleotides in length, siRNAs are dsRNA intermediates that then pair up with a protein complex called the RNA-induced silencing complex or RISC. As the siRNA duplex is unwound, one strand called the “guide strand” is incorporated into RISC while the other strand, called the “passenger strand,” is selectively eliminated. The elimination (i.e., cleavage of the targeted transcript) is done by the “slicer” component of RISC called Argonaute-2 (Ago2). AAV-mediated delivery and liposome-mediated delivery remain the most common forms of siRNA delivery but several other exciting approaches are being actively developed (82).

To date, there are no human studies of RNA interference for the treatment of epilepsy. However, animal studies show some promise. *De novo* mutations in dynamin-1 (*DNM1*) cause a severe DEE with both infantile spasms (IS) and LGS, that is highly resistant to current ASMs (89). Prior to the association of pathogenic human variants in *DNM1* and severe epilepsy, a spontaneous missense mutation in the mouse ortholog, called “fitful,” had already been associated with epilepsy, and fitful homozygotes exhibit a DEE-like phenotype with developmental delay and severe seizures (89). A *DNM1*-targeted microRNA delivered by self-complementary AAV9 into fitful homozygous mice led to a decrease in the seizure phenotype and improved cellular features on brain histology. As hopeful as this initial study is, more work is needed since causative mutations in *DNM1* in humans are usually *heterozygous* dominant negative, in contrast with the *homozygous* mouse model in this study. But as was discussed previously, RNAi is a good approach to envisage when dealing with dominant negative alleles that need to be silenced while maintaining normal expression of the healthy allele.

##### CRISPRi/a

Another approach to gene activation and inhibition relies on nuclease-deficient (or catalytically dead or deficient) dCas9 protein, in conjunction with various effector domains such as transcriptional activators to activate (called CRISPRa) or transcriptional inhibitors to inhibit (called CRISPRi) gene expression (83). Briefly, this process involves conversion of Cas9 protein from a DNA “scissor” into a gene activator by disrupting its nuclease activity. This is done by introducing mutations into the evolutionarily-conserved nuclease domains (RuvC and HNH) of Cas9 to make dCas9, essentially converting the protein into an RNA-guided DNA-binding protein. From there, dCas9 can be fused with an effector (i.e., a transcriptional activator such as VP64 or VPR, or a transcriptional repressor such as the Kruppel-associated box domain of Kox1). The resulting dCas9-effector fusion, when paired with a target-specific single guide RNA (sgRNA) then operates as an artificial transcription factor to activate or inhibit gene expression.

CRISPRa/i approaches to monogenic epilepsy have only been attempted in animal models but appear promising. A CRISPRa approach in a mouse model of DS led to significant decrease in febrile seizures compared to the wildtype control mice, but did not completely suppress all seizure activity (90). A similar result was obtained by a different team using a CRISPRa methodology delivered *via* AAV (91). Both studies suggest that upregulation of *Scn1a* in inhibitory neurons leads to phenotypic improvement, even after the juvenile murine stage, opening a path for future therapies along these lines (90, 91). On a more cautious note, and has been noted in other reviews, these Cas9-based treatments do require long-term expression of exogenous Cas9 proteins in neurons, potentially leading to off-target immunogenic effects (72).

## Conclusion

As genetic causes of epilepsy continue to be discovered, it will be important for clinicians and researchers to leverage genetic and molecular insights to produce precision therapies for these patients. This review highlighted the many ways that monogenic epilepsies can be treated in a targeted manner based on pathophysiological insights. Areas of ongoing promising research include phenotype-treatment studies meant to delineate the specific roles of genetic variants and advances in gene therapies including RNAi, ASO, and CRISPRa/i. Another important area of investigation is discovering the degree to which seizure burden contributes to developmental delays in these syndromes that commonly present as DEE—i.e., does the reduction of seizure frequency lead to an improvement in the developmental aspects of the condition (7, 31, 40)? As personalized therapies improve, the answer to this question will hopefully emerge.

## Author Contributions

VZ wrote the article. CK and BM reviewed and corrected the article. All authors contributed to the article and approved the submitted version.

## Conflict of Interest

The authors declare that the research was conducted in the absence of any commercial or financial relationships that could be construed as a potential conflict of interest.

## Publisher's Note

All claims expressed in this article are solely those of the authors and do not necessarily represent those of their affiliated organizations, or those of the publisher, the editors and the reviewers. Any product that may be evaluated in this article, or claim that may be made by its manufacturer, is not guaranteed or endorsed by the publisher.
